# Long-Focusing Device for Broadband THz Applications Based on a Tunable Reflective Biprism

**DOI:** 10.3390/mi14101939

**Published:** 2023-10-18

**Authors:** Giancarlo Margheri, Tommaso Del Rosso

**Affiliations:** 1 Institute for Complex Systems of National Council of Researches of Italy, Separate Location of Sesto Fiorentino, Via Madonna del Piano, 50019 Sesto Fiorentino, Florence, Italy; 2 Department of Physics, Pontifícia Universidade Católica do Rio de Janeiro, Rua Marques de São Vicente, Rio de Janeiro 22451-900, Brazil; tommaso@puc-rio.br

**Keywords:** thermo-optical devices, long-focusing devices, THz radiation

## Abstract

THz radiation has assumed great importance thanks to the efforts in the development of technological tools used in this versatile band of the electromagnetic spectrum. Here, we propose a reflective biprism device with wavelength-independent long-focusing performances in the THz band by exploiting the high thermo-mechanical deformation of the elastomer polydimethylsiloxane (PDMS). This deformation allows for achieving significant optical path modulations in the THz band and effective focusing. The surface of a PDMS layer is covered with a gold thin film acting as a heater thanks to its absorption of wavelengths below ~500 nm. An invariance property of the Fresnel integral has been exploited to experimentally verify the THz performances of the device with an ordinary visible laser source, finding excellent agreement with the theoretical predictions at 1 and 3 THz. The same property also allowed us to experimentally verify that the reflective biprism focus has a longitudinal extension much greater than that exhibited by a benchmark convex cylindrical mirror with the same optical power. The device is thermo-mechanically stable up to a heating power of 270 mW, although it might be potentially exploited at higher powers with minor degradation of the optical performances.

## 1. Introduction

The radiation in the THz range (0.1 THz up to 10 THz) has so far demonstrated high flexibility for use in many different applications. Indeed, the implementation of tools for bio-medical [1,2,3,4,5], industrial [6,7], communications [8,9,10], and sensing [11,12] applications operating in this frequency range has received great attention, together with the prerequisite of the proper management of THz radiation, and many innovative solutions have been examined so far [13,14,15,16,17,18,19].

Among the different optical functions, long-focusing by means of the generation of quasi-Bessel beams (QBBs) is one of the most studied because of the potential benefits in many fields, like remote sensing, optical trapping, and sensing [20]. Currently, the most common approaches, like Computer Numerical Control (CNC), injection molding, milling [21,22,23], and ultrashort pulsed laser ablation [24], are still time-consuming and costly. Aiming to overcome these drawbacks, recent progresses in 3D printing have been successfully exploited to produce less expensive and even exotic geometries for THz devices [25,26,27,28,29], but at the moment, the resolution of conventional 3D printers (~100 μm) limits the quality of the finishing of the surfaces, and as a consequence, 3D-printed devices usually operate at wavelengths no lower than 500 μm.

More sophisticated devices based on all-dielectric metasurfaces can be effective in generating QBBs [30], but the design tolerances limit the wavelength range of operation and the focus extension and require sophisticated fabrication hardware. Good results have been achieved with the use of more conventional fabrication tools. For instance, traditional polymeric refractive conical axicons exhibited good long-focusing properties in an extended THz range (0.1–1 THz) [31], while axicons working at 0.55 THz formed by the sedimentation of SiO_2_ particles have been demonstrated to reduce the number of fabrication steps [32].

Apart from the inherent drawbacks of complex technical implementation and the potential limitations in the exploitable frequency range, all the proposed devices lack an important feature: tunability at different working wavelengths.

This problem has been partially resolved by the technology of parallel-plate waveguide structures (PPWGs) in the range of 0.1–1 THz [33,34] because the relatively long wavelength considerably relaxes the opto-mechanical tolerances and allows mass production with minimal efforts. This technology has been proposed to fabricate devices operating in a restricted frequency range, i.e., 0.35 THz–0.45 THz, that can be tuned by the simple repositioning of triangularly shaped metal foils [35]. However, PPWG devices do not seem suitable to operate in the range of higher THz frequencies, as the use of lower wavelengths, down to 30 μm, would strongly limit the mechanical design tolerances and the waveguide losses.

In this work, we report on a tunable metallic optical reflective biprism designed for the band of 1–10 THz that exploits the potential of the thermomechanical devices demonstrated in [36].

The core structure is a Fresnel reflective biprism [37,38,39], generated by the modification of the profile of a gold-coated PDMS layer when heating radiation is focused onto a line. Due to the strong absorption of gold by radiations with wavelengths below ∼500 nm, PDMS expands, and thanks to its high dilatation coefficient, a two-pitch roof-like reflective structure is generated. The maximum swelling amplitude is as high as 164 μm, so that under reflection, a significant optical path difference (OPD) corresponding to the spectral region of THz wavelengths can be obtained. Indeed, we theoretically show that when a Gaussian wave at THz frequency impinges on the device, the reflected light beam spreads into two separate lobes in the far field, while a well-resolved long virtual focus is formed behind the reflective surface. It is worth noticing that in the present case, the nondiffracting beam is produced by a 2D biprism rather than by an axicon, so the centrosymmetric QBB generated by axicon-like devices is translated into a nondiffracting cosine beam, representative of the produced long-focus light distribution ([40,41] and references therein). By exploiting an invariance property of the Fresnel diffraction integrals, we implemented a method to experimentally simulate the behavior of the device at 1 and 3 THz by using visible radiation. The proposed reflective biprism can be easily modulated by simply varying the pump power, it operates with low power consumption, and it is not affected by chromatic dispersion in the whole THz band, which represents a common drawback in traditional THz radiation beam-shaping systems.

## 2. Theoretical Modeling

### 2.1. Thermomechanical Model

In this section, we present the modeling of the thermal–mechanical–optical behavior of the proposed device. First, we calculated the maximum continuous-wave (CW) optical power that allows the safe use of the device and then evaluated the swelling profiles at different heating powers. These profiles have been used to predict the optical behavior, with both ray-tracing and electromagnetic calculations.

**Figure 1:**
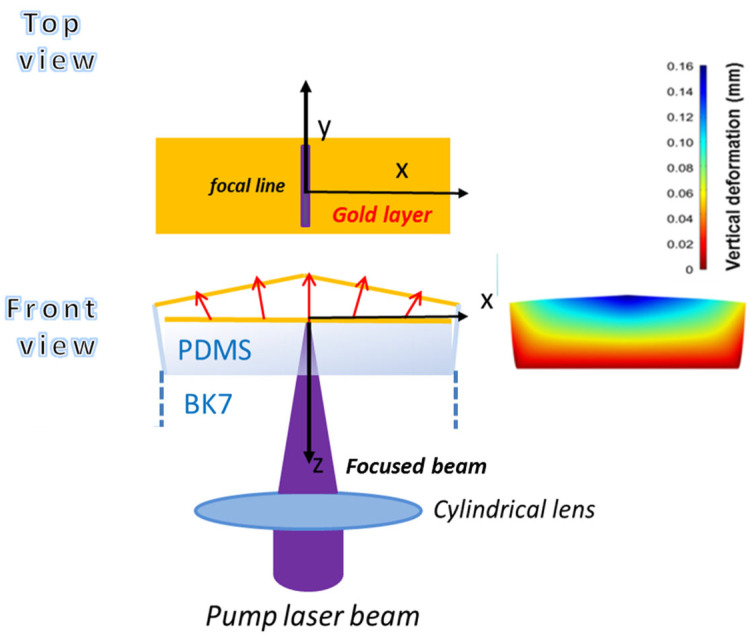
Generation and geometry of the reflective biprism device. Upon the absorption of an astigmatically focused laser beam by the gold coating, the structure heats and the PDSM swells, producing a reflective biprism structure.

The geometry used for the computations is illustrated in Figure 1 and considers three main regions. The lower one is a BK7 glass substrate (1 mm thickness), covered by a layer of PDMS with a thickness of 1.5 mm (second region), onto which a gold thin film with a thickness of about 100 nm is deposited. The device is surrounded by the third medium, which in the present case is air.

The sample is illuminated from the air side with a pump beam (*λ* = 450 nm) that is focused with uniform intensity on the gold layer into a line by a converging cylindrical lens. The heated area has a length of 9 mm, much higher than its transversal FWHM in the *x*-direction of 0.15 mm. Given the huge difference between these dimensions, the thermomechanical behavior of the device can be modeled considering an indefinite 2D structure along the *y*-direction. Since gold has a strong absorption at the wavelength of 450 nm, it efficiently heats the PDMS, provoking its huge swelling due to the high coefficient of linear thermal expansion of about 3 × 10^−4^ °C^−1^ [42].

The purpose of the thermo-mechanical study is to determine the swelling profile at increasing heating powers *P_h_,* particularly the fraction of the optical pump power converted into heat, and establish the power *P_h_* that produces a temperature *T_max_ =* 300 °C, here considered the maximum tolerable temperature. At first glance, this limit can seem excessive, as the data sheets report a temperature limit for PDMS of ~200 °C. Nevertheless, it has been demonstrated that PDMS begins to degrade heavily at temperatures around 300–350 °C, where the components of the polymer begin to decompose into volatile products [43].

**Figure 2:**
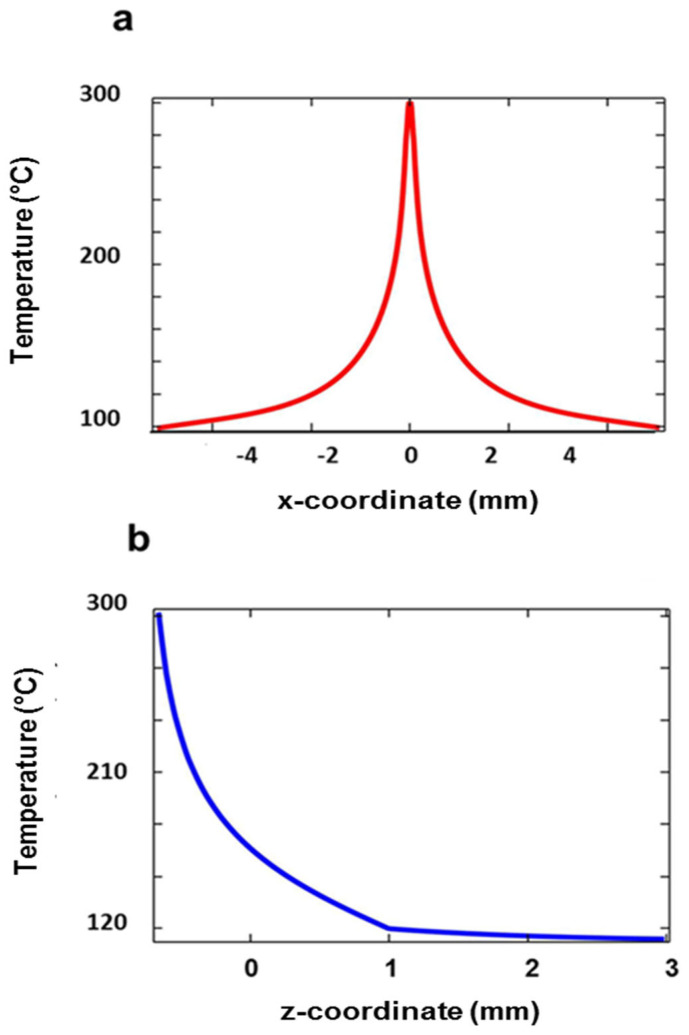
Temperature distribution for the maximum heating power P_hmax_ = 270 mW. (**a**) Surface profile in the *x–y* plane and (**b**) depth profile in the *z*-direction.

The calculations were performed with COMSOL 5.3a software in the 2D indefinite frame. The temperature *T_max_ =* 300 °C is obtained at *P_hmax_ =* 270 mW, corresponding to a power density of 2.0 × 10^5^ W/m^2^. This condition gives rise to the spatial temperature distributions shown in Figure 2a and Figure 2b for surface and depth profiles, respectively. The surface temperature halves at a distance from the symmetry plane of 1.20 mm (*x*-direction), while this occurs at a depth of 1.08 mm from the PDMS surface (*z*-direction). It is worth noticing the huge difference between the transversal dimension of the thermal source and the width of the temperature distribution. This difference is due to the high thermal conductivity of the gold layer (320 W/m·K against 0.2 W/m·K of PDMS) that favors the surface heat flow and the corresponding broadening of the temperature distribution, whose cuspidal-like trend is, however, consistent with the heat source concentration in a sharp sub-mm transversal dimension.

**Figure 3:**
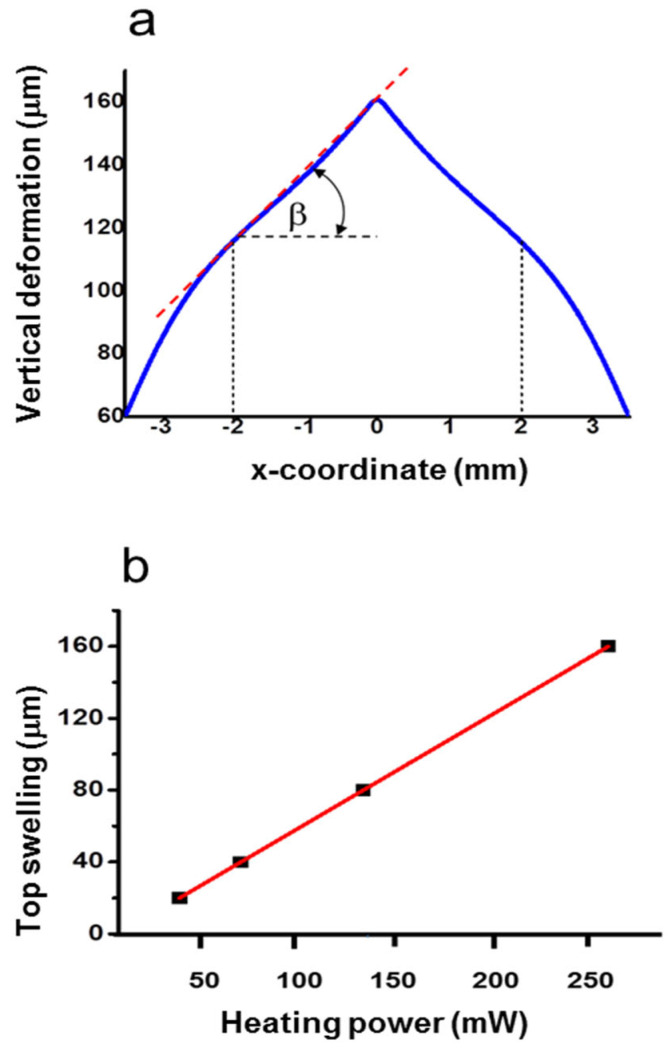
(**a**) Swelling profile of PDMS in the *z*-direction for the heating power of 270 mW. (**b**) Linear trend of the maximum deformation at *x* = 0 mm vs. the heating power.

The temperature T_max_ corresponds to the maximum swelling of 164 μm. The transverse profile of the deformation is reported in Figure 3a. As shown, the profile is described by a *y*-invariant triangle-like function, with almost constant slopes at both sides up to an *x*-coordinate of about ±2.5 mm. Indeed, the root mean square (RMS) of the deviation with respect to the best-fitting linear function is 623 nm with 2 mm on the x-axis. This structure resembles a 1D biprism working in reflective mode. The effect of the increase in *P_h_* is illustrated in the plot of Figure 3b. The top swelling increases linearly with *P_h_*, with a rate *A* = 0.61 μm/mW. Thus, the function representing the *y*-invariant swelling profile, namely *f*(*x*), can be obtained by multiplying an adimensional function *g*(*x*) by a factor H representing the maximum deformation for a given *P_h_*, i.e., *f*(*x*) = *H·g*(*x*), where H = *A·P_h_*. The average slope of the flanks, *β*, increases linearly as well, with a rate of 9.3 × 10^−5^ rads/mW, reaching a value of 0.025 rads at *P_h_ = P_hmax_.* To summarize, the swelling induced in the PDMS layer can be very high, with protrusions that can reach values falling in the THz wavelength range, where a significant spatial modulation of the light intensity is thus expected.

In order to assess the dynamic behavior of the device, we performed preliminary calculations to check the theoretical response times. We found that a time of at least 200 ms is necessary to swell the PDMS structure; thus, it cannot be recommended for use as a dynamic tool, but rather as a static device with a sub-second adjustment time.

### 2.2. Optical Modeling

**Figure 4:**
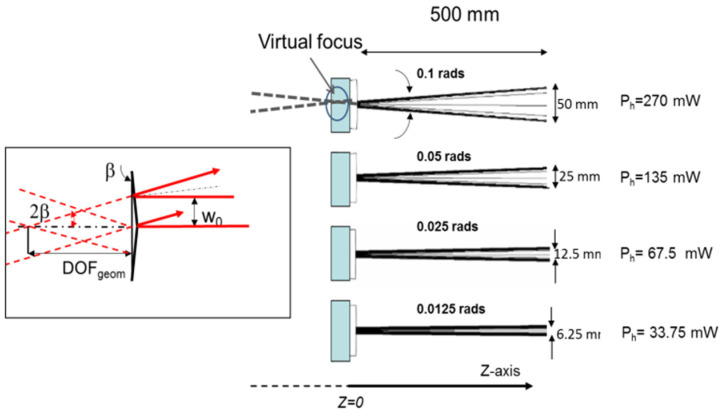
Right side: Ray-tracing simulations illustrating the splitting of the input ray distribution and the formation of the virtual focus behind (z < 0) the deformed surface at different heating powers *P_h_*. Left side: Expanded view of the virtual focus region. *β*: Base angle of the reflective biprism. *DOF_geom_:* Geometrical optics definition of the depth of focus of the device. w_0_ is the beam waist of the impinging Gaussian beam (see text for the mathematical details).

A preliminary evaluation was performed with 2D ray-tracing analysis by coupling three physics modules in Comsol: Heat Transfer, Solid Mechanics, and Geometrical Optics. The input ray distribution is constituted by a bundle of rays with a full aperture of 2 mm, indefinite in the y-direction, impinging on the device parallel to the *y–z* symmetry plane (zero angle of incidence). The results are shown in Figure 4, in which the input rays (not reported in the figure for the sake of clarity) are split into two separate bundles of rays, whose angular aperture is proportional to the impinging power. As 2*β* is the angle of the reflected rays with the axis, the complete aperture is 4*β*, which reaches the value 4*β ~* rads at *P_h_ =* 270 mW.

By prolonging the reflected rays in the region z < 0, a virtual focus is produced. Thus, a positive optical relay will be necessary to effectively use the THz probe radiation. In principle, one could also produce a real focus by launching the probe rays from the PDMS side, but in this way, the radiation would experience a dispersive effect, leading to chromatic aberrations in the considered THz broad spectral window.

Despite the finite extension of the actual input field in the *y*-direction, we will demonstrate in the experimental section that this modeling assumption is adequately reproduced experimentally. With this hypothesis, we calculated the length (*DOF_bp_*) and the minimum width (*D_bp_*) of the virtual line focus. Here, we adopt the definition of *DOF_bp_* as the FWHM extension of the axial light intensity distribution, and *D_bp_* as the minimum FWHM of the intensity distributions onto the *x–y* plane. The field output after the reflection by the reflective biprism facets is calculated with the Fresnel diffraction formula for a cylindrical wave:

(1) $$U(x_{0},z)=\frac{\exp(ikz)}{\sqrt{\lambda z}}\int_{-\infty }^{\infty }U_{ax}(x)\exp(i\pi \frac{x^{2}+x_{0^{2}}}{\lambda z})\exp(-ikx_{0}\frac{x}{\lambda z})dx$$

where *x* is the transverse coordinate in the observation plane at z-coordinate and *U_ax_*(*x*) is the distribution of the field at the reflective biprism output plane, obtained by multiplying *U_in_* for a phase shift due to the axicon reflection (*x*). The field at the axicon plane output *U_ax_*(*x*) is given by

(2) $$U_{ax}(x_{out})=\exp(ik\Phi (x_{out}))\exp(-{\left(\frac{x_{out}}{w_{0}}\right)}^{2})$$

with

(3) $$\Phi (x)=4\pi \frac{AP_{h}g(x)}{\lambda }$$

In the hypothesis of thin optical element, we can assume *x_out_ ~ x,* obtaining

(4) $$U(x_{0},z)=\frac{\exp(ikz)}{\sqrt{\lambda z}}\int_{-\infty }^{\infty }\exp(-{\left(\frac{x}{w_{0}}\right)}^{2})\exp(4\pi i\frac{g(x_{out})AP_{h}}{\lambda })\exp(i\pi \frac{x^{2}+x_{0^{2}}}{\lambda z})\exp(-ikx_{0}\frac{x}{\lambda z})dx$$

In this case, the intensity is given by:

(5) $$I(x_{0},z)=\frac{{\left|U(x_{0},z)\right|}^{2}}{\lambda z}$$

where *I*(*x*_0_,*z*) is the power per linear unit in the *x*–*z* plane.

An inspection of Equation (4) shows that the integrals depend on the product *λz* and the ratio *A·P_h_*/*λ* rather than on the wavelength alone. This fact suggests an interesting way to infer the light distribution at a given wavelength *λ*_1_ and using a heating power *P_h_*_1_, if the behavior at another wavelength *λ*_2_ and heating power *P_h_*_2_ are known.

We can indeed introduce the following scaling rules:

(6) $$\lambda_{1}z_{1}=\lambda_{2}z_{2}$$

(7) $$\frac{P_{h1}}{\lambda_{1}}=\frac{P_{h2}}{\lambda_{2}}$$

where *λ*_1_ and *λ*_2_ are two wavelengths of the probe beams impinging on the reflective biprism, generated with top swellings *A·P_h_*_1_ and *A·P_h_*_2_, respectively, and *z*_1_, *z*_2_ are the corresponding z-coordinates at which the fields are calculated. It is easy to verify that under the conditions described by Equations (6) and (7), the integral of Equation (4) does not change. This property will be used in the experimental section to retrieve the light distribution at THz frequencies from measurements performed at a visible frequency.

**Figure 5:**
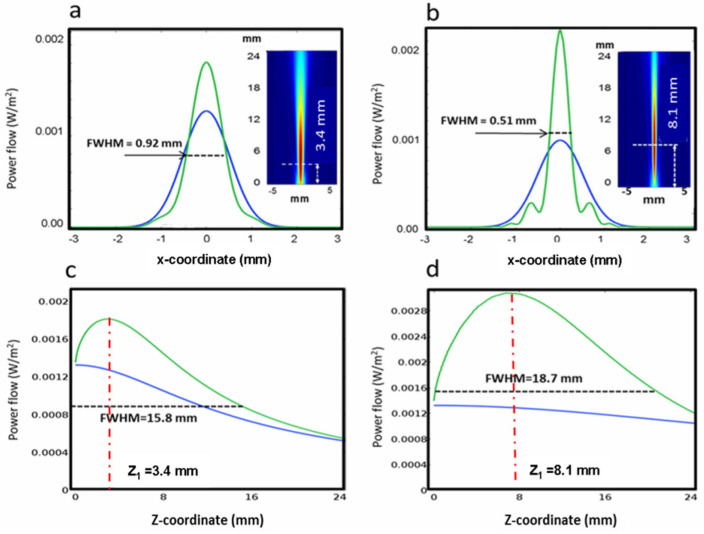
Intensity profiles for *P_h_* = 270 mW (green curves) and *P_h_* = 0 mW (blue curves). (**a**,**b**) Calculated transverse intensities in the focal points at 1 THz (focus distance: 3.4 mm) and 3 THz frequency (focus distance: 8.1 mm), respectively. (**c**,**d**) Corresponding axial intensities.

The modification of the light distribution due to the significant phase changes at THz frequencies, just previously anticipated, are clearly visible in the plots of Figure 5, reporting the focused intensity distributions at 1 and 3 THz radiations. The occurrence of the focusing effect is clearly visible. Indeed, the maximum intensity increases with respect to the maximum Gaussian input intensity *I*_0_ without pumping (blue lines) from approximately 30% at 1 THz to about 100% at 3 THz frequency (green lines). This focusing becomes rapidly less efficient at lower frequencies. For instance, at 0.6 THz (0.5 mm wavelength), the maximum intensity in the focus lowers to 16%. As is well known, increasing the focus intensity for a fixed probe input *w*_0_ and optical power is feasible only by increasing the base angle *β* [44], with an increased risk of overheating. However, we will show in the experimental section that this occurrence may not actually be so detrimental to the device performances if their minor worsening can be tolerated.

On the contrary, the focusing becomes more efficient at higher frequencies. In order to cover the higher THz band, we repeated the calculations for 5 THz and 10 THz. The minimum width *D_dp_* passes from 0.92 mm at 1 THz to 0.42 mm at 5 THz and 0.20 mm at 10 THz frequency, while the focus intensity increases to 257% (5 THz) and 370% (10 THz) with respect to *I*_0_. The *DOF_bp_* becomes nearly constant at ~20 mm with increasing frequency (see Table 1). It is worth noticing that this last result is consistent with the geometrical optics predictions. Indeed, referring to the scheme on the left side of Figure 4, the geometrical depth of focus is approximately given by *DOF_geom_* = *w*_0_/2*β* mm, which resembles the calculated *DOF_dp_* with improved approximation at decreasing wavelengths, as expected in the geometrical optics limit. In order to assess the focusing advantages of this device, we can compare these results with those achievable by using conventional cylindrical optics. This comparison gives meaningful information primarily about the improved depth of focus of the proposed reflective biprism device. To this end, we considered a convex cylindrical mirror with a focal length *f_mir_* equal to the coordinate *z* of maximum intensity of the reflective biprism (hereafter the reflective biprism focal length *f_bp_*) and the same Gaussian input, i.e., *U_in_*(*x*) *=* exp(*−*(*x*/*w*_0_)^2^), and calculated the corresponding *DOF_G_* of the mirror with the well-known relations of Gaussian optics. In particular, the focused virtual beam waist *w*_1_ is calculated with the relationship *w*_1_*= f_mir_ λ πw*_0_, where *f_mir_ = f_bp_*, *λ* is the radiation wavelength, *w*_0_ is the waist of the field input (=1 mm), and the depth of focus is *DOF_G_ =* 2*πw*_1_^2^ *λ*, which is twice the Rayleigh range. The results are given in Table 1. At all frequencies, *DOF_bp_* > *DOF_G_*, from 7 times to about 20 times at 1 and 10 THz, respectively, demonstrating the advantage of the reflective biprism in terms of long-focusing with respect to an equivalent optic with the same focal length.

## 3. Experimental Section

We experimentally simulated the optical performances of the thermally generated reflective biprisms at the frequencies of 1 and 3 THz by using a probe laser light in the visible range, i.e., the radiation of a He-Ne laser at the wavelength of *λ*_2_ = 0.633 μm. The proposed approach is justified by the scaling property of the Fresnel integral, as shown in the previous section, and will be detailed later on.

The preparation of the sample was performed as follows. First, a conventional microscope glass slide was covered with a PDMS layer. We accurately mixed the monomer and the curing liquid in a 10:1 volume ratio; then, a volume necessary to generate a 1 mm nominal thickness polymer was poured onto the glass surface. After degassing the liquid solution (about 0.5 h), the monomer was left to polymerize overnight in air without any pre-heating. A thin gold layer (100 nm nominal thickness) was successively deposited at a rate of 0.5 nm/s onto PDMS by thermal evaporation under high vacuum conditions (pressure: 4 × 10^−5^ Torr).

Then, we measured the absorption of the gold layer at the CW pump radiation wavelength of 450 nm, which was 85%. This absorption value is different from 74%, calculated with WINSPALL 3.02 software for a bilayer structure composed of PDMS (refractive index: 1.46) and Au (refractive index: 1.47 + 1.95i) [45]. However, this difference can be attributable to a nanostructuration of the deposition of Au onto the porous PDMS surface which, as is well known, can give rise to substantial differences in the optical properties with respect to a flat deposition [46,47]. At a given *P_in_* pump power, the heating power *P_h_* is thus given by *P_h_ = P_in_·*0.85.

In the second step, we checked the behavior of the swelling vs. the heating power by analyzing the reflected beam pattern. The ray tracing analysis has shown (Figure 4) that the reflected rays form two bundles separated by an angle that is directly proportional to the input power.

**Figure 6:**
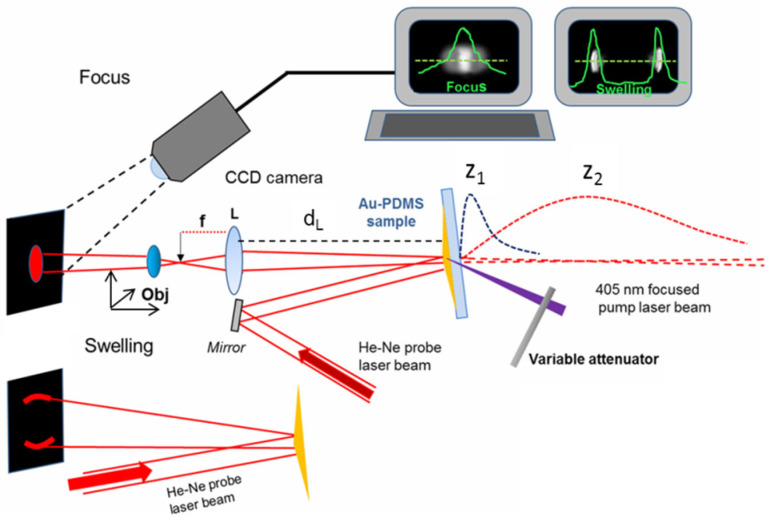
Experimental setup. In the lower part (“Swelling”) is shown the optical layout used to indirectly measure the swelling of the thermally generated Fresnel reflective biprism. The focused pump beam (violet triangle) heats the PDMS, which forms the structure. The probe beam coming from a He-Ne laser (*λ*_2_ = 633 nm) is reflected by the Au-PDMS sample and is left to propagate toward the observation screen. The input beam is divided into two main lobes in the far field, whose inter-distance depends linearly on the heating power *P_h_*). In the upper part (“Focus”), the virtual foci at 1 and 3 THz (λ_1_ = 300 μm and λ_1_ = 100 μm, respectively), whose coordinates are z_1_ = 3.4 mm (1 THz) and 8.1 mm (3 THz), are scaled to *λ*_2_ following condition 2. These fields are reproduced at the same wavelength closely to the focus of a positive lens L (focal length f = 100 mm, distance d_L_ from the sample = 600 mm) and magnified (140×) with an objective Obj (0.65 NA, 40×) to be properly visualized on a screen and recorded by a CCD camera.

Thus, referring to the “Swelling” part of Figure 6, we generated the extrusion of the PDMS surface by using different heating powers, from the minimum required (*P_h_* = 0.58 mW) up to the maximum theoretically allowable for safe use (*P_h_* = 270 mW). The impinging probe beam, coming from a He-Ne laser (*λ*_2_ = 0.633 μm), is reflected towards the screen into two separate lobes. We recorded the intensity profiles at a 500 mm distance from the sample on the transverse axis of the light distribution.

With the “Focus” part of the setup, we simulated, at the probe wavelength *λ*_2_*,* the reflective biprism by focusing the 1 THz and 3 THz radiations. By using the proper pump heating powers calculated from Equation (7), namely *P_h_* = 0.57 mW and 1.74 mW for the simulation of 1 THz and 3 THz, respectively, we generate the devices, which correspond to swellings of 164 μm (1 THz) and 54.5 μm (3 THz). The probe beam waist is expanded from 0.55 mm to 1 mm, impinges on the device, and, upon reflection, produces virtual focused fields that reproduce those at THz frequencies, but with the *z*-coordinates *z_z_* scaled with respect to *z*_1_ at *λ*_1_ (Equation (6)). These fields are demagnified by the lens L and transformed in real images, re-expanded by an objective (140× magnification) on a screen, recorded with a CCD camera, and analyzed in real time with the image-processing software IMAGE PRO Plus^®^6.0. By axially moving the objective, we lock the position *z_max_* of the focus when the maximum intensity is observed. The *x*-intensity distribution is recorded along a transversal line in the central part of the light pattern. The same setup was adopted to simulate the long-focusing performance at 1 THz frequency. However, this is a more difficult operation to perform a priori. Indeed, the transformation of the axial virtual light distribution into the real one is not so direct, because the longitudinal de-magnification of the lens L is not uniform. This demands more considerations to support the measurement strategy, detailed in the Supplementary Materials.

Experimentally, the objective is moved back and forth with respect to *z_max_* of the amount *dz* = 0.3 mm, and the fall in intensity is measured with respect to the maximum by recording the transversal intensity along the same lines as before. This fall is compared to that calculated when the reflective biprism is changed with a fictitious convex cylindrical mirror with the same focal length, and this comparison enables us to establish the advantage of using the reflective biprism with respect to the focusing of a conventional optic.

## 4. Results and Discussion

**Figure 7:**
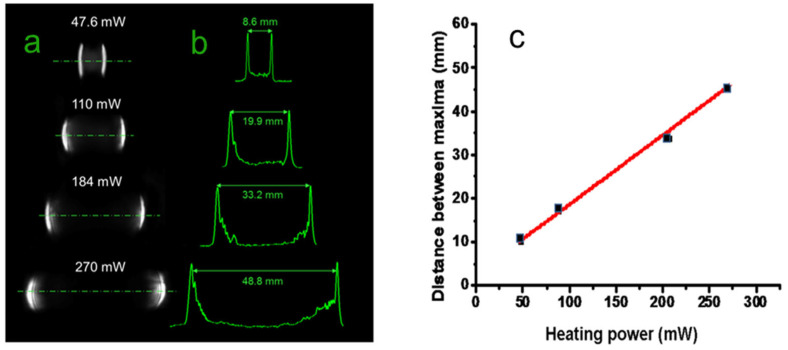
Photographs (**a**) and traces (**b**) of the far-field intensity recorded at 500 mm from the screen for various heating powers. (**c**) Distance between intensity maxima vs. heating power *P_h_*. The wavelength of the probe beam was *λ*_2_ = 633 nm.

The photos of the far-field intensity patterns and their transversal profiles reflected by the reflective biprism device are reported in Figure 7a,b for various heating powers.

There is clear evidence of the splitting of the input beam, which increases linearly with increasing *P_h_,* as reported in Figure 7c, as theoretically predicted. We raised the input power until an effect of surface cracking was evidenced by the upset of scattering light around the far-field pattern, an occurrence observed at *P_h_* = 285 mW, not far from the theoretical maximum value of 270 mW.

**Figure 8:**
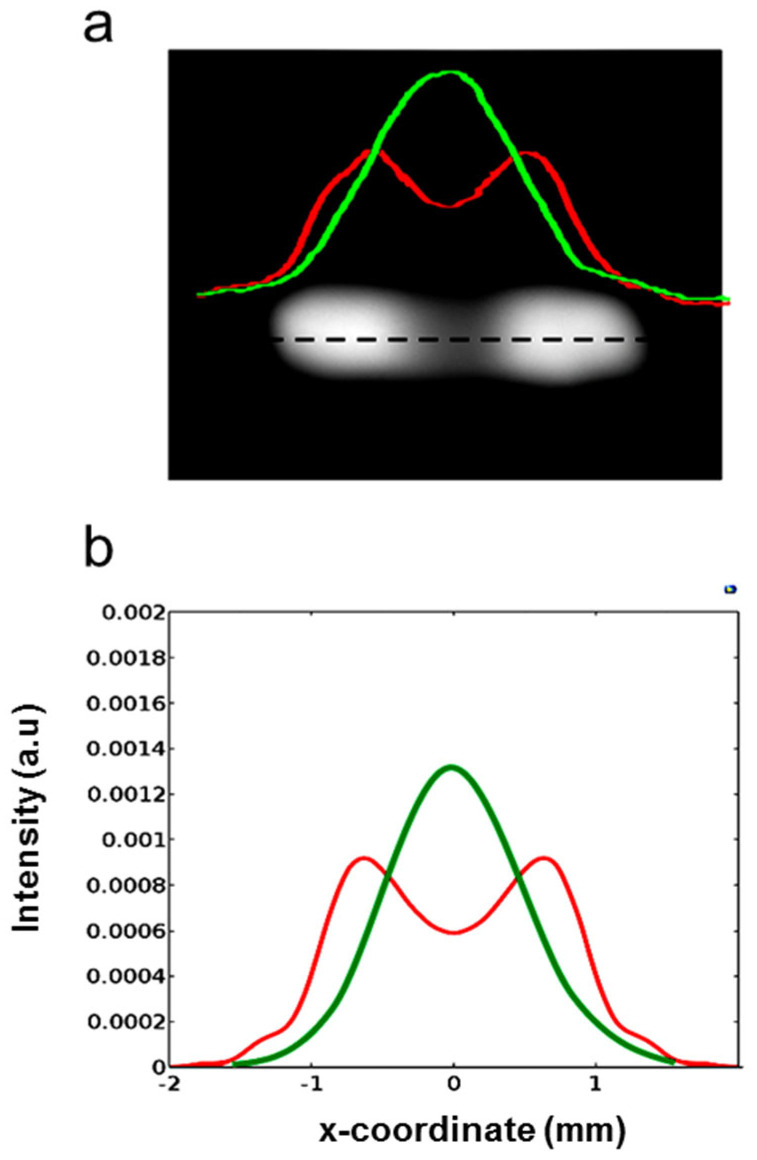
(**a**) Photograph and far−field intensity profiles, recorded on the dashed black line, of the probe beam (*λ*_2_ = 0.633 μm) reflected by the device generated with a heating power *P_hvi_*_s_ = 0.58 mW, at a distance of 1280 mm from the sample. The 1D intensity pattern was recorded on the horizontal symmetry axis of the light distribution (dashed black line) with (red line) and without (green line) the pump light. (**b**) Calculated probe beam in the far field produced at the same distance by the device with a swelling of 344 nm (red line) or without pump power (green line).

The distance between the maxima at this heating power is 48.8 mm, corresponding to a full angle of 0.098 rads, with a rate of increase of 2.8 × 10^−4^ rads/mW. Repeating the measurements in five different heating cycles at the same power, we found that apart from the growth of low-intensity scattered light, due probably to fine roughening of the irradiated area, the main lobes remain practically unchanged. When the pump is OFF, the probe beam spot undergoes only a small geometrical variation, indicating a substantial reversibility of the structure. Of course, in view of practical applications, more checks are needed to validate these results, as thermal degradation and aging of the PDMS are well-known issues that can seriously worsen the structure’s stability. To summarize, the maximum base angle *β* experimentally found with ineffective damage is *β*~0.098/4 = 0.0245 rads (1.4 deg.). The maximum phase change expected at the lower frequency of 1 THz (wavelength *λ*_1_ = 300 μm) for a beam width *w*_0_ of 1 mm is *4πβw*_0_*/λ*_1_~1 rad, and can thus produce significant focusing effects, as theoretically predicted by the electromagnetic calculations shown in the modeling section. Considering the radiation at 1 THz, its simulation at *λ*_2_ requires a swelling of 344 nm, which is 476 times lower than that generated by the highest pump power. Despite this huge difference, the effect of the PDMS swelling is still well resolved. Indeed, as shown in Figure 8, the two lobes are not completely separated in the far field, but nevertheless are clearly formed. Thus, in this case, well-defined focusing should also be experimentally found.

Furthermore, the focusing of 3 THz radiation is expected to be effectively produced.

**Figure 9:**
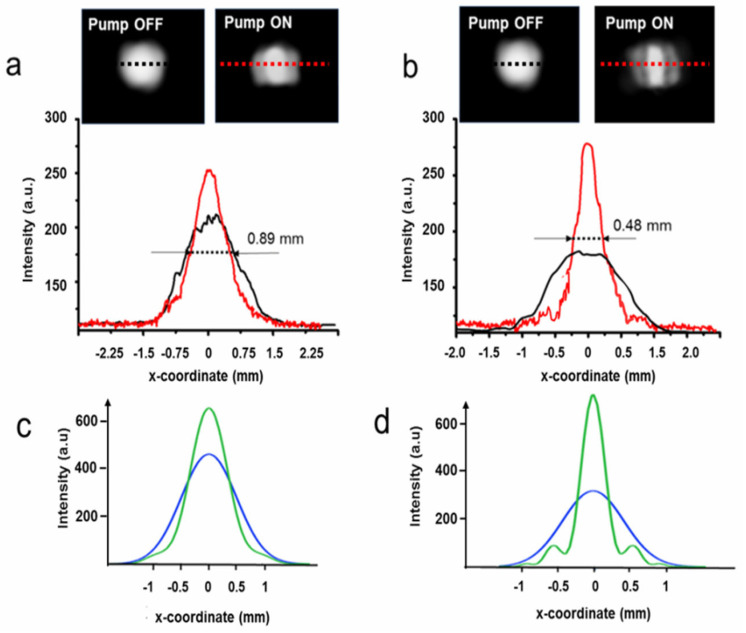
Experimental intensity profiles of the reflected probe beam (*λ*_2_ = 633 nm) measured with the setup of Figure 6. The results simulate the focusing of the reflective biprism at *λ*_1_ = 300 μm (1 THz) and *λ*_1_ = 100 μm (3 THz) in (**a**) and (**b**), respectively. Insets: Photos of the light distributions when the pump is OFF or ON. The experimental light intensities are recorded on the horizontal black dotted lines (no pump power) and red dotted lines (pump power ON) (**c**,**d**). The same profiles of Figure 5c,d are reported here for the reader’s convenience.

The focal transversal light distributions corresponding to the simulations of 1 and 3 THz radiations are reported in Figure 9a,b together with those obtained without the optical pumping (black lines). The measured FWHMs are 0.89 mm and 0.48 mm for the experimental simulations of 1 THz and 3 THz, respectively. The results are in good agreement with the theoretical intensities calculated with the “true” THz radiations, considering the proper heating powers and swellings of the reflective biprism, as the intensity plots reported in Figure 9c,d show that their FWHMs are 0.92 mm (1 THz) and 0.51 mm (3 THz), very close to the theoretical predictions, giving experimental evidence of the affordability of the simulation approach.

**Figure 10:**
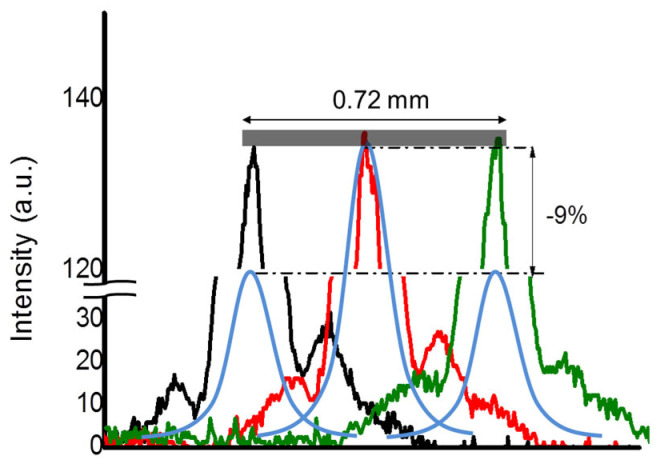
Experimental intensity transversal profiles of the probe beam (*λ*_2_ = 633 nm) in the focal region of the reflective biprism, simulating the 1 THz radiation (black, red, and green traces). The intensity is constant within 1% (gray rectangle) in a range of 0.60 mm, while it should decrease by 9% if the focus is produced by a convex cylindrical mirror with the same focal length of the device (3.4 mm) at 1 THz (pale blue lines). The foci produced by the actual device and the convex cylindrical mirror, which are calculated to actually be 0.22 mm apart, have been superimposed for the sake of clarity.

The results of the long-focusing simulation are represented by the traces of Figure 10, reporting the transversal intensity profiles at the axial points *z_max_*, *z_max_ + dz*, *z_max−_dz.* We found that axial intensity remains constant within 0.8%, which is the resolving power of the experimental detection system, and for this reason, the predicted change of 0.2% did not have clear evidence.

However, the experimental percentage is approximately one order of magnitude lower than the decrease in percentage intensity calculated in the Supplementary Materials for the focusing of an equi-focal convex cylindrical mirror (−9%). This occurrence demonstrates that the reflective biprism that focuses the 1 THz beam is much more robust to diffraction with respect to that calculated for the conventional focusing of a benchmark convex cylindrical mirror, indicating the presence of a much greater depth of focus.

While the substantial stability of the device was observed at *P_h_* = 270 mW, we also checked the modifications induced on the gold surface using heating powers up to *P_hmax_* = 285 mW, at which cracking effects caused strong variations to the intensity pattern.

The reflected probe beam far-field intensity at *λ*_2_ is still constituted by two separate lobes that increase their distance to 98 mm at *P_hmax_*, but compared to the distributions in Figure 7, their light pattern suffers some broadening and smearing towards the center.

**Figure 11:**
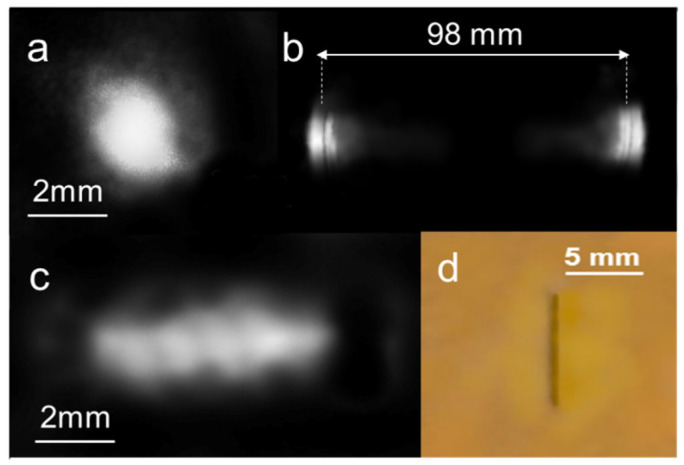
Experimental 2D intensity profile of the reflected probe beam (λ_2_ = 633 nm): (**a**) before the optical pumping; (**b**) with optical pumping, after 5 heating cycles at *P_h_* = 285 mW; (**c**) at zero pump power after 5 heating cycles. (**d**) Photo of the gold surface after 5 heating cycles. The fracture line corresponding to the focal pump irradiation is evident.

**Figure 12:**
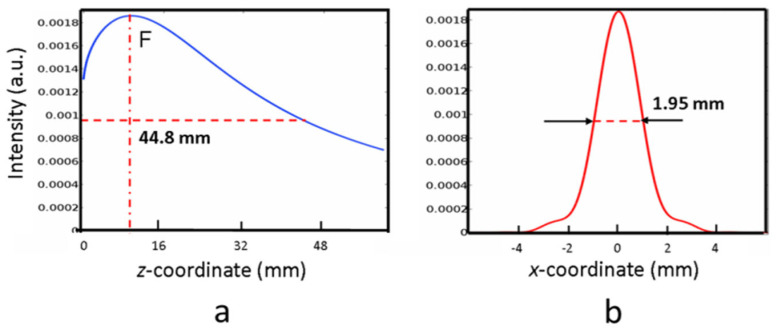
Simulated behavior of the reflective biprism at 0.45 THz (wavelength: 0.667 mm). The waist of the input Gaussian beam is *w_0_* = 2.5 mm; the axicon angle is 0.025 rads. The dashed red lines represent the FWHM of the axial (**a**) and transversal (**b**) light intensities. The distance of the focus F from the surface is 11.8 mm. These theoretical results are comparable to those obtained in Ref. [34]. See text for details.

Moreover, when the pump is switched OFF, the pattern no longer resembles that of the initial Gaussian beam; rather, it appears deformed in a fringed-like figure elongated in the horizontal direction, as visible in Figure 11c. This pattern indicates a crack on the surface, whose edges move apart from each other when the PDMS swells and re-shut at zero pump power. Surprisingly, we found that in five repeated cycles of heating, the far-field intensity pattern develops only a slight intensity nuance towards the center, presumably due to the scattering and diffraction introduced by the crack, resembling the pattern reported in Figure 12b. The stability in the pattern indirectly evidences that the surface crack reaches a steady shape, and that a base angle *β* as high as 0.05 rads (3 deg.) can be reversibly obtained using heating powers that overcome the safe temperature of 300 °C. A check of the gold surface after the thermal cycling actually confirmed the presence of that vertical crack (Figure 11d). As the optical path difference in the reflective biprism is proportional to *β*, significant phase changes in the lower THz band (0.1–1 THz) could still be obtained, presumably without major issues in the focusing performances. The verification of this claim is beyond the scope of the present work and will be reported in future investigations. A possible drawback of this cracked structure is that the underneath PDMS is no longer shielded from the external environment by the gold layer, so degradation issues are more likely to occur in shorter times [48].

To the best of our knowledge, this is the first report on the performances of a Fresnel biprism working in the THz range above 1 THz, so a direct comparison with other reports was not possible. However, the concept of 1D long-focusing in the THz range was successfully demonstrated by using PPWG reflective biprisms working below 1 THz frequency [34]. At a frequency of 0.45 THz, supposing *w*_0_ = 1 mm, our device reaches a theoretical *DOF_ax_*~30 mm, and the minimum measured *D_ax_* is approximately 1.88 mm. However, considering a higher *w*_0_ = 2.5 mm, which is approximately the limit of linearity of our device (see Figure 3a), we found the intensity behavior of Figure 12, where a *DOF_ax_* = 44.8 mm, compared to 46 mm reported in [34], while the FWHM in the focus, 1.95 mm, is only slightly higher than the FWHM of 1.85 mm deducible from the data shown in Figure 3 of [35].

Long-focusing in the band 0.1–1 THz constantly receives noticeable attention. For instance, quite recently [32], the use of refractive conical axicons with base angles of 10° has allowed the generation of a focus spot 7 mm wide in a depth of focus exceeding 160 mm. A similar performance can be reached with our device. Indeed, considering the simulation implemented for 0.45 THz, the spot width of 1.85 mm can be expanded with positive relay optics with transverse magnification *M* = 3.8. Following the magnification law of geometrical optics for axial magnification, its approximate value is M^2^ = 14.4, leading to a theoretical *DOF_ax_*~460.8 mm. This value suggests that our device can reach, at least to the order of magnitude, the high depth of focus reported in [32].

As a final remark, we wish to stress that working in a broader range below 1 THz would require a redefinition of the gold coating according to the used frequency. In fact, in the range 0.1–1 THz, the skin depth of gold becomes higher than 100 nm; thus, the gold layer thickness should be increased to avoid transmission losses versus the underneath dielectric. Even if this increase would produce no detrimental mechanical weakening, experimental work is necessary to assess this statement. From the optical quality point of view, the aberration and scattering issues of the reflecting surfaces are greatly downscaled by the high wavelength even at the highest frequency (10 THz) of the THz range, as suggested by the comparison of the wavelength corresponding to 10 THz frequency, namely 30 μm, and of the RMS of the extruded profile (see Section 2.1).

## 5. Conclusions

In this paper, we have presented an Au-PDMS reflective biprism able to generate a virtual long focus using radiations in the frequency band 1–10 THz. The working principle of the device is based on the swelling of a PDMS layer provoked by heating a gold layer generated by the absorption of focused violet laser light in a tight line. Thanks to the high expansion coefficient of PDMS, the deformed reflective surface of the elastomer generates differences in the optical path of a probe beam that fall into the range of THz wavelengths. The corresponding phase changes are significant and allow us to efficiently shape the impinging probe beam in long foci. The reflective biprism can be fabricated with cost-effective tools and is easily tunable, as its shape can be modified by simply varying the input heating power without any mechanical adjustment. Thanks to an invariance property of the Fresnel integral, scaling rules can be introduced that allow us to experimentally simulate the behavior of the device at 1 and 3 THz frequencies with a probe red beam, thus avoiding the expensive labware necessary for generating the THz radiation and its management. The experimental results are in good agreement with the theoretical predictions and confirm the effectiveness of the device in the long-focusing of THz radiation. As the reflective biprism produces a virtual focus, a relay optic is necessary to use it in practice. If from one side this represents an obvious drawback, it can be easily circumvented by coupling a conventional concave mirror to form a real long focus. Thanks to the high wavelengths of the THz radiation, such an all-catoptric device would have minor concerns for optical aberrations, while chromatic effects are completely absent. As the current device has limited performances below 1 THz, our future research will aim at investigating the improvement of the device stability and the focusing performances in the band 0.1–1 THz by exploiting the experimentally observed reversible extra-swelling of the gold surface reflector beyond the cracking limit.

## Figures and Tables

**Table 1:** Comparison between the theoretical focal depth of the reflective biprism produced with a heating power *P_h_* = 270 mW, focal length *f_bp_,* and a convex cylindrical mirror with focal length *f_mir_ = f_bp_*.

Frequency (THz)	*f_bp_* (mm)	*DOF_bp_* (mm)	*DOF_G_* (mm)
1	3.4	15.8	2.2
3	7.1	18.7	3.1
5	8.0	19.0	2.44
10	6.1	19.2	0.94

## Data Availability

Not applicable.

## Supplementary Materials

The following supporting information can be downloaded at: https://www.mdpi.com/article/10.3390/mi14101939/s1, Figure S1: (a) Schematic representation of the simulation at the wavelength λ_2_ = 0.633 μm of the focus region produced by the axicon at 1 THz frequency (flattened blue triangle). The length *dz* (=0.30 mm) is an arbitrarily small width of the longitudinal region around the point of maximum intensity, placed at *z_max_* = 3.4 mm (blue dashed quadrilateral). The virtual expanded simulation region is represented by the red dashed quadrilateral. (b) Transfer and demagnification in the real space by the lens L (focal length = 100 mm) of the expanded virtual focal region. The variation ΔI_ax_ at the endpoints of the focal region is compared with that one, ΔI_cyl_, calculated when the axicon is changed with a cylindrical mirror with the same focal length.
