# Supplementary material for: Long-Focusing Device for Broadband THz Applications Based on a Tunable Reflective Biprism
Source: Micromachines (Basel). 2023 Oct 18;14(10):1939. doi: 10.3390/mi14101939 (PMC10609471; doi:10.3390/mi14101939)
Supplement: Supplementary file 1 [file micromachines-14-01939-s001.zip › micromachines-2644387-supplementary.pdf]

Supplementary material

In this section we design a procedure based on the use of visible radiation (wavelength λ_2 = 0. 633 µm), to simulate the longitudinal focusing performances of the axicon at 1THz frequency (wavelength λ_1 =300 µm). Referring to the scheme of Fig. S1 , the axicon produced with a heating power of 270 mW (swelling : 164 µm) , focuses virtually the radiation at 1 THz frequency with a maximum intensity I_{max} at a distance z_{max}= 3.4 mm from the surface, with a focus $FWHM_x$ = 0.92 mm.
Let's consider a segment of length $2\delta z$ centered at z_{max}, at the ends of which the relative intensity reduction is $\Delta I/I_{max}$. As explained in the Theoretical modeling section, by properly reducing the heating power we can generate an axicon that yields at a wavelength λ_2 the same spatial field distribution produced at the wavelength λ_1. Indeed, the field at coordinates $z_{max}\pm\delta z$ is reproduced at unchanged transversal coordinates, but at expanded z-coordinates, namely $M\ (z_{max}\pm\delta z) = M\cdot z_{max}\pm\ \delta\zeta$, where $\delta\zeta = M\cdot\delta z$ =142.8 mm and $M=\lambda_1/\lambda_2$= 476. The maximum intensity is transferred at the point P_F whose z-coordinate is $z_F = z_{max}M$ and here the $FWHM_x$ remains unchanged while its distance from L is $p_F = d_L + z_F$ = 2218 mm and the overall D_{ax} is enlarged to 7520 mm. This expanded virtual focal region at λ_2 is imaged and demagnified in the real space by the positive lens L .

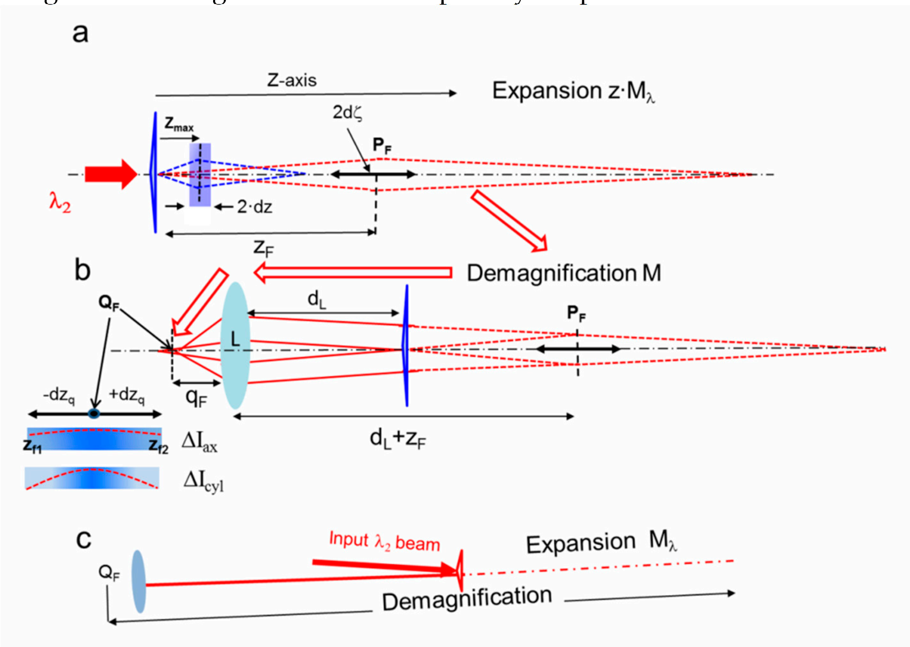

Figure S1. (a) Schematic representation of the simulation at the wavelength λ_2 = 0.633 µm of the focus region produced by the axicon at 1 THz frequency (flattened blue triangle). The length dz (= 0.30 mm) is an arbitrarily small width of the longitudinal region around the point of maximum intensity, placed at z_{max}= 3.4 mm (**blue dashed quadrilateral**). The virtual expanded simulation region is represented by the **red dashed quadrilateral** . (b) Transfer and demagnification in the real space by the lens L (focal length =100 mm) of the expanded virtual focal region. The variation I_{ax} at the endpoints of the focal region is compared with that one, ΔI_{cyl}, calculated when the axicon is changed with a cylindrical mirror with the same focal length.
(c) In order to simplify the optical set-up, in the experimental layout the two sections (a) and (b) are collinear and tilted by a small angle with respect to the input light probe at wavelength λ_2 . For the detailed description see the text of Appendix.

However , aiming to consider the imaging of the whole focus region, one should take into account that the demagnification suffers a strong change along the axis, and as a consequence the light intensity distribution downline of L would have strong distortions with respect that one in the axicon focus of 1 THz radiation. In order to avoid this complication and simplify the calculations, we will select $\delta\zeta \ll p_F$ by choosing an arbitrarily low value of $\delta z = 0.3$ mm $\ll z_{max}$ in our case. By applying the law of conjugate points, P_F is imaged in the point Q_F at a distance $q_F = p_F/M = 104.72$ mm from L, with a transversal de-magnification $M = 22.2$. As it can be easily verified, M is uniform within ±3.5 % with respect to its average value ($M_{av} = 21.8$) in the segment centered on P_F, whose length is $2d\zeta$. As $\delta z/p_F = 0.064 \ll 1$, the length δz_q, longitudinal image of $d\zeta$, can be simply found by applying the approximate relationship of geometrical optics $\delta z_q \sim \delta\zeta/M^2 = 0.29$ mm. The z-coordinates of the endpoints of the focal region, centered at distance q_F, are thus $z_{f1} = (q_F+\delta\zeta)/M^2$, $z_{f2} = (q_F-\delta\zeta)/M^2$. As M is almost constant, the virtual field in the expanded focal region is transferred in the real focus with almost uniform M-scaling in the transversal coordinates and M^2 -scaling in the axial coordinate. As a consequence, indicating with I_{max} the maximum intensity in the focus Q_F, the ratios of the intensities at its endpoints, $I(z_{f1})/I_{max}$ and $I(z_{f2})/I_{max}$, reply with good approximation the ratios of the intensities at the virtual endpoints of the expanded focal line at λ_2 and, for the scaling property of Eq.5, at the endpoints of the simulated focal line at 1 THz. To summarize, the axial intensity distribution generated by the axicon at 1 THz frequency can be reproduced with good fidelity by using the visible probe beam at λ_2, provided that only a small region around the focus is considered. It is transformed in a region close to the focus of the lens L that is demagnified $M=22.2$ in the cross section and $M^2=492$ in the axial direction. The intensity fall at the endpoints ΔI_{ax} is $\Delta I_{ax} \sim 0.2\%\ I_{max}$, so the intensity is almost uniform in the considered width around the focal point. Even if we limited ourselves to examine the intensity variations in a small region around the focus, this is still enough to evaluate the long focusing performances if we compare this result to that one achieved if a conventional focusing optics would be used.
To demonstrate this statement, we replace the axicon with a fictitious convex cylindrical mirror that focuses virtually at the coordinate z_{max} the same Gaussian beam used as input for the axicon (beam waist $w_0 = 1$ mm) . The focal distance f_{THz} of the mirror is thus $f_{THz} = z_{max}$. The beam waist at focus is $w_1 = \lambda_1 f_{THz}/\pi w_0 = 0.32$ mm. Then, as for the axicon, we change the wavelength λ_1 with the simulation wavelength λ_2. The focused field is calculated with Eq.5 by simply substituting the phase factor corresponding to the axicon (Eq.3) with that one introduced by the fictitious optics, that is $\Phi(x) = \pi x^2 P_1/\lambda_1$, where $P_1 = 1/f_{THz}$ is the dioptric power of the mirror. This expression of $\Phi(x)$ has a form analogous to that one of Eq.3 that describes the phase variation introduced by the axicon, namely a constant multiplied for a function of x. Thus, the replication at λ_2 of the field distribution produced at λ_1, is possible by expanding the z-coordinate to zM_λ and changing the power of the mirror to $P_2 = P_1/M_\lambda$. The focal length is enlarged to $f_2 = M_\lambda f_{THz} = z_F$, while the virtual focus waist w_1 is simply translated to z_F . The lens L provides to transfer this focus in the real space. The well known formulas of Gaussian optics permit to calculate the beam waist of the focused beam and its distance from L, that result $w_f = 0.0148$ mm and 104.50 mm respectively. The corresponding Rayleigh range is $z_f = \pi w_f^2/\lambda_2 = 0.98$ mm. The relative intensity fall with respect to the maximum I_{max} expected at coordinates $\pm dz_q$ from w_f , namely $\Delta I_{cyl}/I_{max}$, can be approximately calculated by the formula $\Delta I_{cyl}/I_{max} \sim 1-(1+(dz_q/z_f)^2)^{-1} = 9\%$, to be compared with the observed constant trend of the axial intensity vs. distance.
